# OaAEP1 Ligase-Assisted Chemoenzymatic Synthesis of Full Cysteine-Rich Metal-Binding Cyanobacterial Metallothionein SmtA

**DOI:** 10.1021/acs.bioconjchem.3c00037

**Published:** 2023-03-15

**Authors:** Anastasiia Antonenko, Avinash Kumar Singh, Karolina Mosna, Artur Krężel

**Affiliations:** Department of Chemical Biology, Faculty of Biotechnology, University of Wrocław, F. Joliot-Curie 14a, Wrocław 50-383, Poland

## Abstract

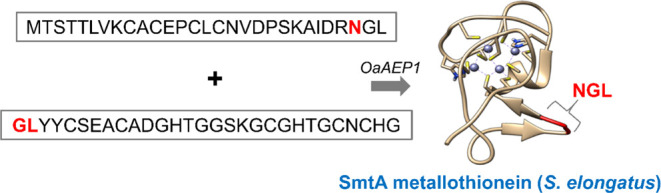

Among all approaches used for the semisynthesis of natural or chemically modified products, enzyme-assisted ligation is among the most promising and dynamically developing approaches. Applying an efficient C247A mutant of *Oldenlandia affinis* plant ligase OaAEP1 and solid-phase peptide synthesis chemistry, we present the chemoenzymatic synthesis of a complete sequence of the cysteine-rich and metal-binding cyanobacterial metallothionein Synechococcus metallothionein A (SmtA). Zn(II) and Cd(II) binding to the newly synthesized SmtA showed identical properties to the protein expressed in *Escherichia coli*. The presented approach is the first example of the use of OaAEP1 mutant for total protein synthesis of metallothionein, which occurs in mild conditions preventing cysteine thiol oxidation. The recognition motif of the applied enzyme could naturally occur in the protein structure or be synthetically or genetically incorporated in some loops or secondary structure elements. Therefore, we envision that this strategy can be used for efficiently obtaining SmtA and for a wide range of proteins and their derivatives.

## Introduction

Protein semisynthesis constitutes a dynamically developing field of biochemistry. It relies on combining synthetic and recombinant peptide fragments in a controlled manner to acquire functional biomolecules, which have wide applications in many areas.^1−3^ Therefore, the development of new and efficient production strategies is of great interest and practical importance. The basic approaches to peptide and protein synthesis are focused on the chemical condensation of peptide precursors coupled with amino acids into longer polypeptide chains. Along with the development of protecting groups that mask the side chains of the amino acids and protect them from reactive coupling conditions,^4^ the ability to assemble them in a user-defined manner in the complete peptide chain immobilized on an insoluble porous support (solid-phase peptide synthesis, SPPS) makes the chemical synthesis of polypeptides easily attainable.^5^ It is reminiscent of ribosomal synthesis and consists of iterative coupling steps, such as anchoring, deprotection, coupling reaction, and cleavage, which could be automated. However, despite the technical feasibility of automation, its speed, and flexibility, it suffers from being laborious and of low efficiency to routinely prepare peptides longer than 40 amino acid residues, considerably lower than the average size of protein domains, peptide therapeutics, or vaccines, e.g., chemokines or histones.^1,6−10^ A similar problem concerns the condensation reactions of fully protected synthetic peptides, which are extremely difficult to manipulate in both aqueous and organic solutions. This is where native chemical ligation (NCL) of an amide bond between unprotected protein fragments can play a role.^11−13^ Native peptide bond ligation is based on the condensation of the C-terminal thioester as an “active ester” with the second peptide or protein with the N-terminal cysteinyl residue in mild conditions (neutral pH) with no side products.^14^ Currently, NCL application allows for the synthesis of proteins and their fragments, whose length reaches 300 or even more amino acid residues.^14,15^ Importantly, it also allows the ligation of two different protein building blocks containing various decorations by post-translational modification,^16^ fluorophores,^17,18^ non-coded amino acids,^19^ functional groups for bio-orthogonal modifications,^20,21^ selective metal-mediated protein heterodimerization,^22^ or for studying the structure and functions of protein complexes.^23^ But like all other methodologies, NCL also has its limitations. Since NCL is a chemical reaction, it requires high concentrations of both the reactants, the presence of α-thioesters, and thiol derivatives to induce thiolysis, which restrict its application to many proteins.^24^ Also, there are no available reports for the synthesis of MTs by NCL.

Aside from pure chemical approaches, protein engineering has at its disposal other alternative methods based on enzymes, e.g., sortase,^25−29^ butelase,^30,31^ and OaAEP1,^32−37^ which demonstrate highly selective and site-specific reactivity. Compared to other strategies, the enzymatic methods are characterized by good efficiency, compatibility with a wide range of biological molecules, and the possibility of carrying out reactions under mild conditions.^38,39^ Nonetheless, their time-consuming necessity, special coenzyme requirements, and sometimes low catalytic efficiency necessitate constant research on applied biocatalysts, their improvement for semisynthetic protein manipulation, or a combination of different approaches to use their benefits effectively. In this article, we describe protein semisynthesis which combines the benefits of SPPS chemical peptide synthesis with enzymatic ligation through the transacylation of amines. One of the new and attractive enzymes in protein modifications is Asx ligase (Asn/Asp asparaginyl endopeptidase, OaAEP1) isolated from the *Oldenlandia affinis* plant and now expressed in the *Escherichia coli* system in an active form.^33^ Its natural role is the synthesis of plant cyclotides,^34^ and it constitutes an excellent alternative to the enzymes present in the current protein engineering toolkit. It is due to the requirement of a short tripeptide C-terminal recognition motif, NGL, and a faster transpeptidation rate.^1^ Moreover, its improved variant, the OaAEP1_C247A mutant, is 160 times more active than the wild type, which makes it an attractive tool for protein semi- or total synthesis.^36^ Furthermore, it has been shown that the enzyme can operate the substrates not only in the regular N-to-C orientation but also in C-to-C ligations,^40^ which enlarges the synthetic scope of the enzyme. Despite the unique properties of OaAEP1 and its improved version, this enzyme has not yet been applied for the chemoenzymatic synthesis of any full-length protein.

**Figure 1:**
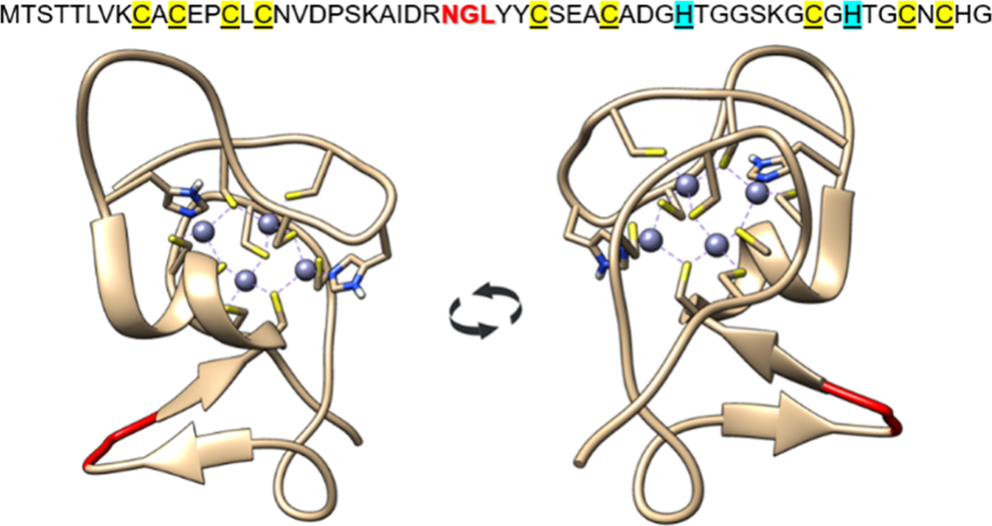
Cyanobacterial MT SmtA. Top: amino acid sequence of SmtA (Uniprot ID: P30331) with the native NGL motif indicated in red. Yellow and blue underlined letters indicate cysteine and histidine residues participating in metal ion binding, respectively. Bottom: X-ray structure of SmtA (PDB: 1JJD). The NGL motif is marked in red. Gray spheres represent Zn(II) ions clustered in the Zn_4_Cys_9_His_2_ core.^46^

In the present work, we focus on the chemoenzymatic synthesis of one of the representatives of the cyanobacterial metallothionein group—Synechococcus metallothionein A (SmtA) from Synechococcus PCC 7942, which is responsible for the metal storage, transport, and homeostasis in the cell.^41^ So far, the most common method for the production of metallothionein (MT) and MT-like small Cys-rich proteins is their recombinant production using the *E. coli* system.^42,43^ Even then, the production yield varies significantly for different MTs since Cys-rich proteins are toxic for bacteria due to essential metal ion chelation or the cellular redox potential being affected.^44^ Cyanobacterial SmtA, similar to other MTs, binds essential Zn(II) and Cu(I) ions and efficiently sequesters Cd(II).^41^ SmtA was found, for example, to protect cells against Zn(II) toxicity^43^ and control many cellular processes, such as DNA synthesis, translation, and transcription.^45^ Its NMR structure indicates the formation of a Zn_4_Cys_9_His_2_ clustered core demonstrating structural similarities to other (in)vertebrate, plant, or bacterial MTs and some zinc finger domains (Figure 1).^43,44,46,47^ Overall, the approach described here may solve some problems of synthesizing proteins rich in Cys residues or their post-translational modifications, which can be introduced during chemoenzymatic synthesis.

## Results and Discussion

**Figure 2:**
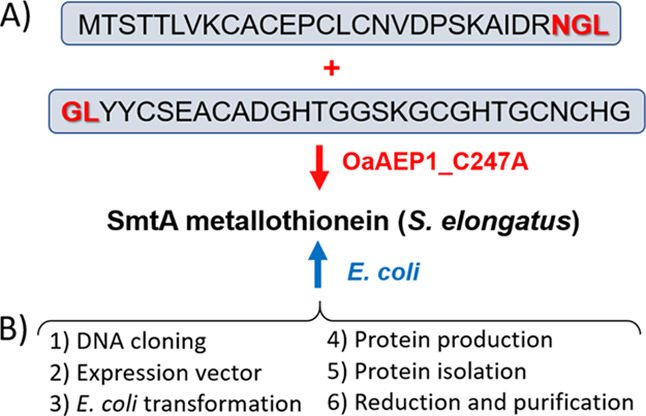
Two methods of SmtA protein production used in this study. (A) New one based on a single step of chemically synthesized fragments of SmtA and further ligation with enzymatically improved OaAEP1_C247A. (B) Production of SmtA in the *E. coli* bacterial system required multiple steps.

The molecular target of this work is a 56-amino acid-long low-molecular-weight MT from the cyanobacteria *Synechococcus elongatus* (SmtA). It has been chosen for OaAEP1-assisted chemoenzymatic synthesis due to the presence of its recognition motif—NGL sequence (Figures 1, 2A), as well as the presence of a high number of cysteine residues. As a reference, the aforementioned MT has also been independently overproduced in *E. coli* (Figure 2B).

**Figure 3:**
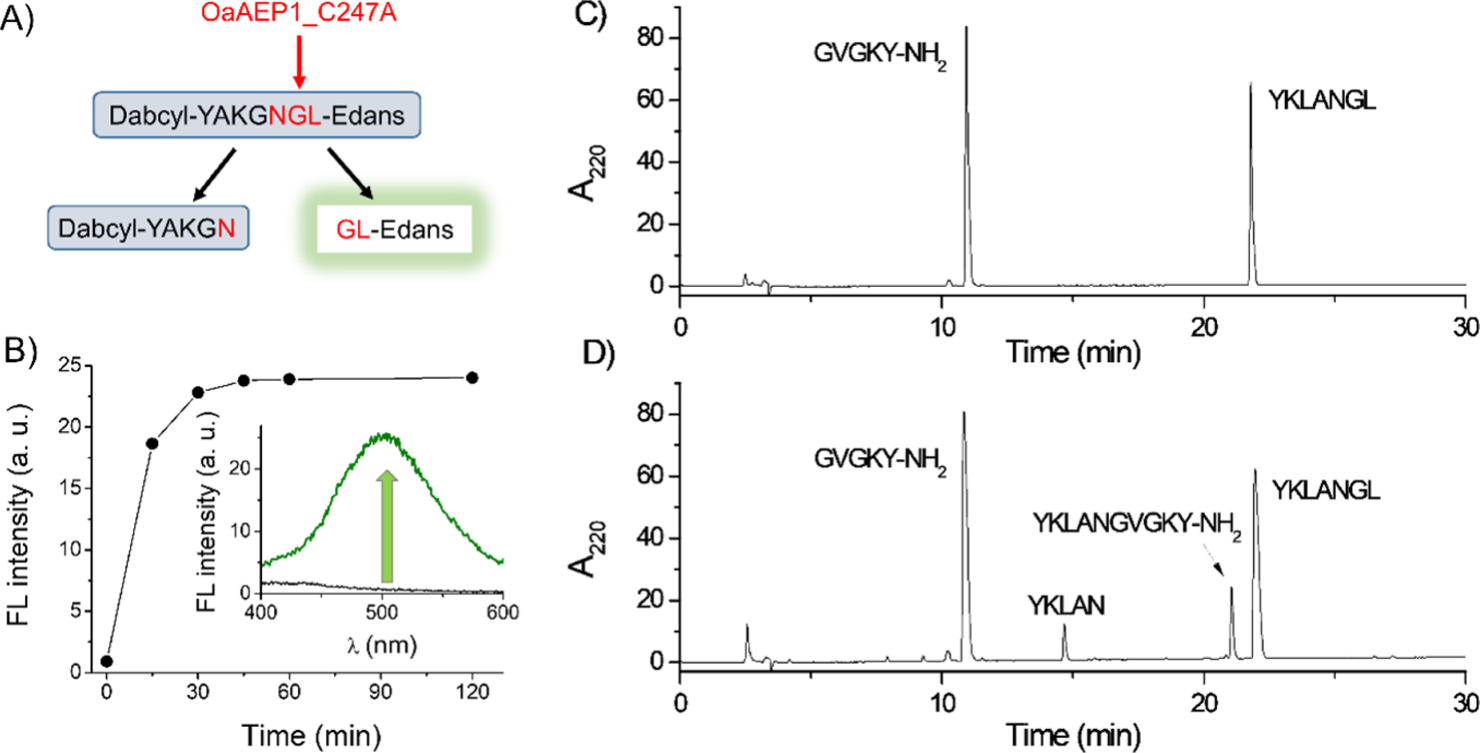
Fluorometric and RP-HPLC activity assay for the OaAEP1_C247A used in this study. (A) Ligase fluorometric activity was carried out using the specific FRET substrate Dabcyl-YAKGNGL-Edans. The substrate was hydrolyzed (reverse to the ligation reaction), and the characteristic fluorescence of GL-Edans at 490 nm was measured when it was excited at 340 nm. (B) Fluorometric reaction kinetics was monitored for 120 min using 0.5 mM Dabcyl-YAKGNGL-Edans in the presence of 25 μM OaAEP1 C247A at 37 °C. Inset indicates emission spectra at 0 and 120 min timepoints. a.u. denotes arbitrary units. (C) RP-HPLC profile for the model peptides, 0.25 mM N-terminal YKLANGL, and 0.5 mM C-terminal GVGKY-NH_2_ using OaAEP1 ligase. (D) RP-HPLC profile for the ligation reaction of model peptides, a peptide mixture of 0.25 mM N-terminal YKLANGL and 0.5 mM C-terminal GVGKY-NH_2_ with 25 μM ligase monitored during the reaction at 37 °C after 2 h. YKLAN-OH is the hydrolysis product. Peptides (substrates and products) were separated on a C18 column (250 mm × 4.6 mm, 5.0 μm) in the gradient of 5–35% of MeCN in 40 min and 35–85% of MeCN in the next 20 min, and absorbance was recorded at 220 nm.

In the first step, we expressed and purified the Cys247Ala mutant of OaAEP1 in *E. coli* and stored it at −80 °C (Figure S1 and Table S1). Since the activation of this plant enzyme is not trivial, we examined its activity after this process using the FRET Dabcyl-YAKGNGL-Edans substrate. The nonfluorescent peptide turns into the fluorescent GL-Edans (λ_ex_ = 340 nm, λ_em_ = 490 nm) product only when the enzyme is active (Figure 3A,B).^48^ Because this test is based on substrate hydrolysis, ligase activity has also been examined using model peptides containing recognition termini, YKLANGL and GVGKY-NH_2_. The ligation reaction was monitored by analytical high-performance liquid chromatography (HPLC) (Figure 3C,D) and indicated the appearance of YKLANGVGKY-NH_2_, the right product of the reaction, which confirms the enzymes’ activity. Besides the desired ligation product, the hydrolyzed YKLAN peptide was also detected (Figure 3C, Table S1). It is because the final product represents the NGV motif that constitutes a substrate for asparaginyl endopeptidase, which results in product hydrolysis. Additionally, another model nucleophile, GLGKY, was also tested, and the ligation result was positive (Figure S6).

**Figure 4:**
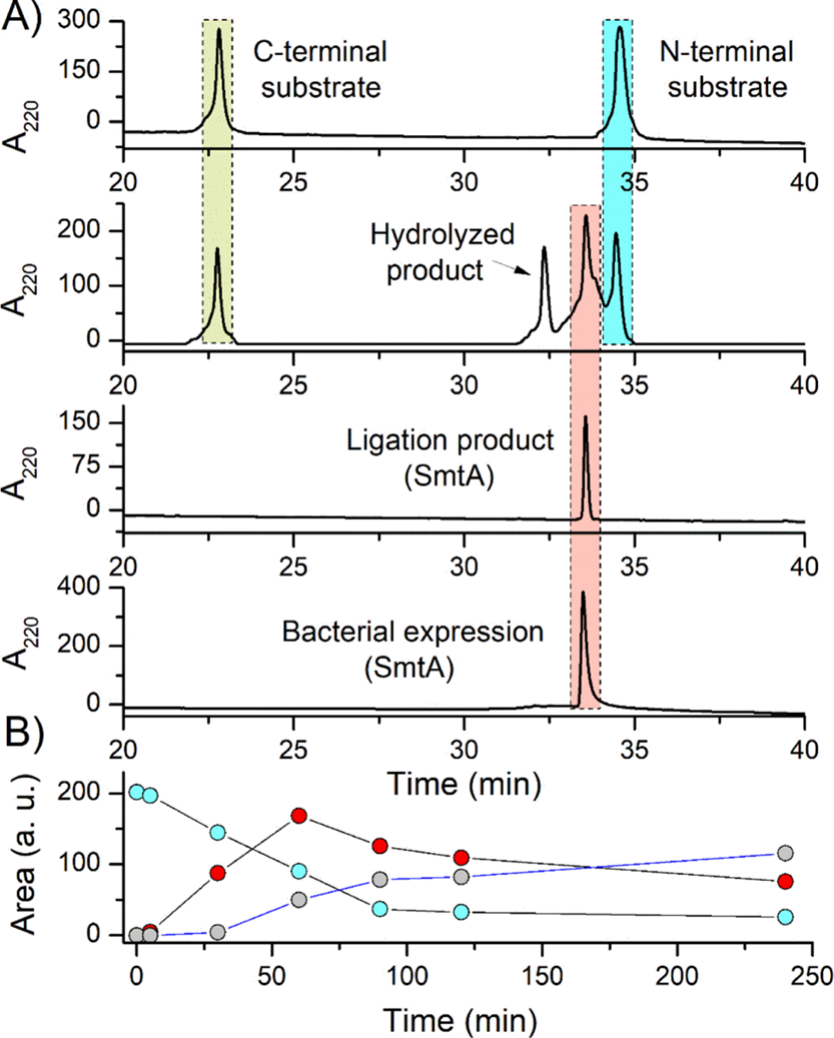
RP-HPLC of OaAEP1-assisted synthesis of SmtA. (A) Chromatograms of substrates before the addition of the enzyme, elution profile after 60 min; purified synthetic SmtA product, and bacterial SmtA (see below). The ligation mixture contains 0.5 mM N-terminal and 0.25 mM C-terminal substrates with 100 μM ligase. (B) Time-dependent decay of the N-terminal substrate (light blue circles) and formation of its hydrolyzed product (gray circles) and SmtA MT (red circles).

We then directed our attention to ligating the two peptidyl substrates for the target SmtA semisynthesis. They were selected in such a way that both contained OaAEP1 recognition motifs (represented by NGL and GL sequences) (Figures 2A and S3) and were synthesized according to the Fmoc strategy on a solid support, cleaved from the resin, purified using reversed-phase (RP)-HPLC, and lyophilized (Figure S3, Table S1). For the ligation reaction, both C- and N-terminal peptides were mixed in sodium acetate buffer, pH 5.6, at a molar ratio of 0.5. Then, OaAEP1 was added, and reaction progress was monitored by HPLC for 240 min (Figure 4A). In this case, similar to model peptide ligation, besides the expected SmtA product (red color), the hydrolyzed one was also formed during the reaction (blue color). The largest fraction of the SmtA product was observed after 60 min of the reaction, while the hydrolyzed product reached its highest content after 100 min (Figure 4B). In order to increase the yield, the reaction was simply optimized regarding the substrates’ molar ratio, and the reaction time was tested from 5 to 180 min. It showed that the incubation of C- to N-terminal substrate molar ratio for 0.5 and 1 h at 37 °C resulted in more than 70% product formation (Figure S2). The almost linear correlation between N-substrate utilization and product appearance observed for the first 60 min becomes no longer proportional because of the reversibility of the reaction and is accompanied by the appearance of the hydrolyzed product (Figure 4B). The HPLC retention time and molecular mass for the ligation product (synthetic SmtA) were found to be 33.8 min and 5740.22 Da, respectively, which were similar to those of the bacterial-expressed SmtA (*t*_R_ = 33.5 min, 5740.77 Da) (Figure 4, Table S1).

**Figure 5:**
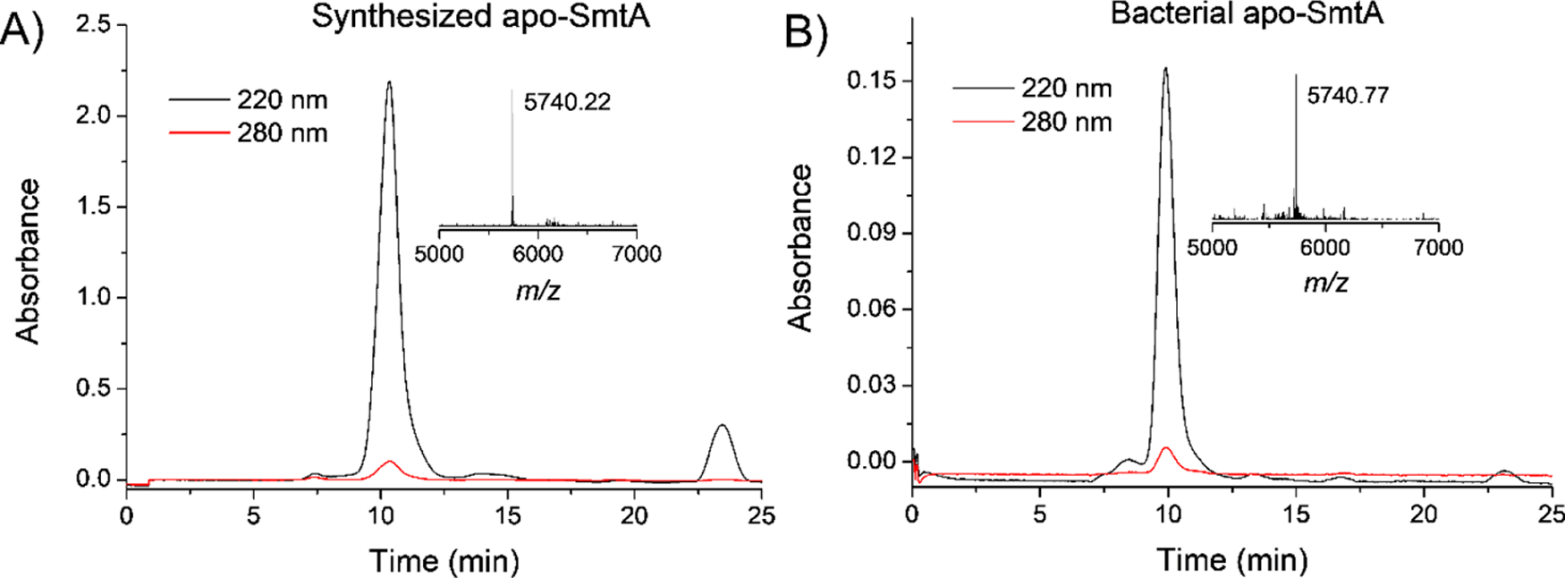
SEC purification of synthesized (A) and bacterial SmtA (B) on a Superdex Peptide column (10/300 GL) using 10 mM HCl, and absorbance was recorded at 220 and 280 nm.

Due to the fact that HPLC analysis indicated successful SmtA ligation, we proceeded to the essential stage of our work, namely, the semi-preparative synthesis of MT. The scale of synthesis was increased 10 times, and the reaction was carried out in buffer [50 mM sodium acetate pH 5.6, 50 mM NaCl, 1 mM ethylenediaminetetraacetic acid (EDTA), and 0.5 mM tris(2-carboxyethyl)phosphine hydrochloride (TCEP)] for 60 min at 37 °C. Due to the scale of the reaction, the mixture was separated at pH ∼ 2 to avoid thiol oxidation using size exclusion chromatography (SEC) with a Superdex Peptide column (10/300 GL) (Figure S4).^49^ All observed signals were identified, the final apo-SmtA product was separated, and its purity was confirmed in a separate analysis (Figures 5, S4). After SEC, 407 μg (200 μL of 354.40 μM; yield = 70.5%) of the apo-SmtA product was obtained with more than 95% purity (Figure 5). The comparison of the reaction yield of the presented method with other ligation approaches is not possible because there is no available report regarding the chemical synthesis (NCL or SPPS) or semi-synthesis of MTs. To confirm the retention time of apo-SmtA and subsequently metal-binding properties, bacterial SmtA was also produced in the *E. coli* system as presented in Figure 2B and described in detail in the Experimental Procedures. Its SEC retention time was 10 min and the molecular mass was 5740.77 Da, which were similar to those of the synthetic protein, 10 min and 5740.22 Da, respectively (Figure 5, Table S1).

**Figure 6:**
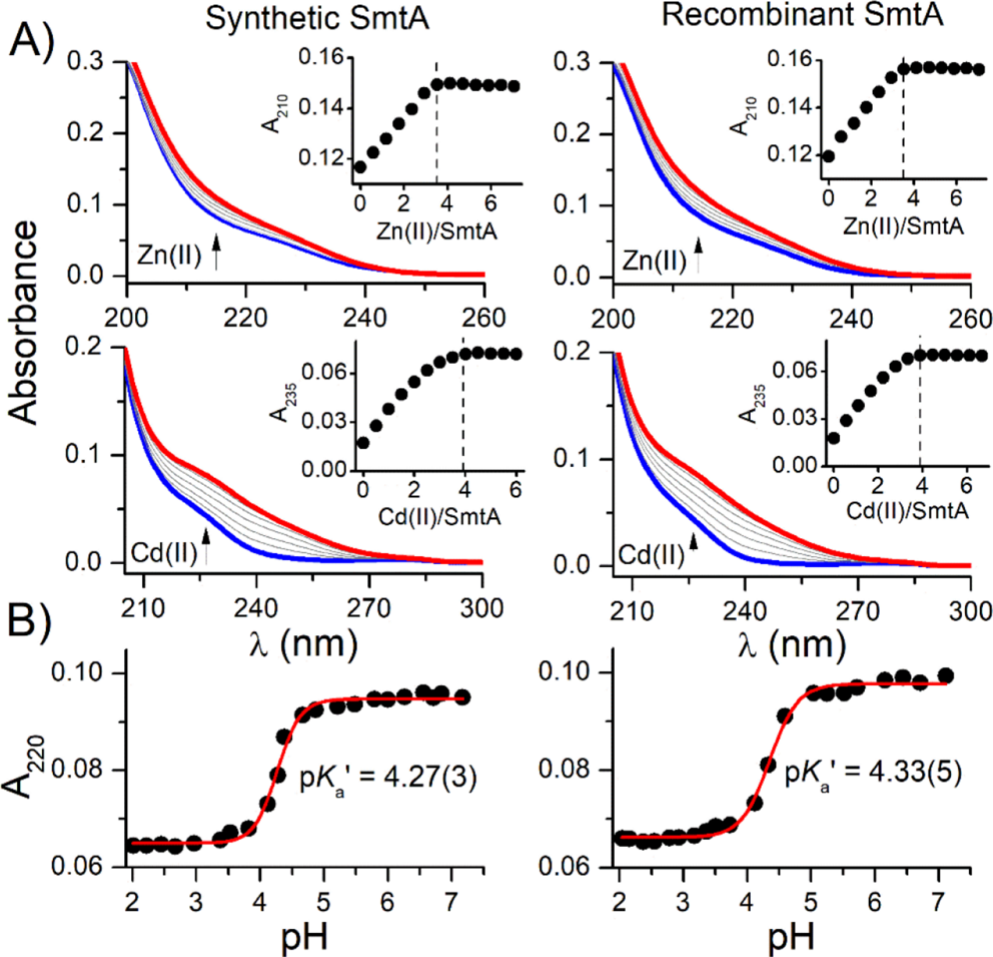
Comparison of metal-binding properties of chemoenzymatically synthesized and expressed in bacteria SmtA. (A) Spectroscopic titration of apo-SmtA with Zn(II) (top row) and Cd(II) (medium row) in 50 mM borate buffer pH 7.4 (50 mM NaCl) with 45 μM TCEP. The insets demonstrate the increase in absorbance at 210 and 235 nm for Zn(II) and Cd(II), respectively. (B) pH-dependent absorption increase of apo-SmtA mixed with Zn(II) at a molar ratio of 1:4.2 monitored at 220 nm. Data were fitted to Hill’s equation.^51^ Standard deviations of the last digit are given in parentheses.

To compare the binding properties of apo-SmtA when chemoenzymatically synthesized and when produced in *E. coli*, it was spectrophotometrically titrated with Zn(II) and then with Cd(II). Since both metal ions bind to thiolate sulfurs, characteristic LMCT (ligand-to-metal charge transfer) bands are observed for Zn(II) (∼200 to 240 nm) and Cd(II) (∼210 to 270 nm) (Figure 6A). Differential spectra demonstrating more clear LMCT bands are presented in Figure S5. For both proteins, electronic spectra are highly similar to each other, and absorption increases at chosen wavelengths up to ∼3.5 and 4 equiv of Zn(II) and Cd(II) over apo-SmtA, respectively, and becomes constant or slightly decrease, as reported previously.^46^ Interestingly, differential spectra obtained for Zn(II) titrations show a much more significant maximum hypochromic band shift than for Cd(II) (Figure S5), which indicates differences in the metalation pathway during the formation of the M_4_Cys_9_His_2_ core, which is a reason for Zn(II) saturation at ∼3.5 equiv instead of 4 equiv. Similar trends were recently reported for human MT2, indicating differences in the cluster formation for Zn(II) and Cd(II).^50^ The CD spectrum of synthetic SmtA indicates proper folding compared to the bacterial one (Figure S7). The chromophoric properties of the metal–thiolate complexes provide a means to assess their stability by pH titrations. Therefore, assuming identical acidity of all cysteine thiols, we determined average p*K*_a_’ values of thiol groups in the presence of Zn(II) for both types of SmtA.^51^ Note, that the p*K*_a_′ value differs from p*K*_a_ for spontaneous thiol deprotonation. That value represents metal-to-protein affinity, and it is often used for stability comparison among various metalloproteins and metal–peptide complexes.^52−54^ Experimentally calculated p*K*_a_′ values for synthetic and recombinant SmtA proteins are 4.33 ± 0.05 and 4.27 ± 0.04, respectively (Figure 6B), and are convergent with the previously reported value of ∼4.2.^55^ Their high similarity together with the same spectroscopic and stoichiometric properties indicate that chemoenzymatically synthesized SmtA MT demonstrates identical metal-binding properties as those produced in bacteria, indicating that the method presented here can be used for the preparation of similar cysteine-rich proteins as well as other biomedically important proteins.

## Conclusions

In summary, this work presents a new OaAEP1-mediated approach for complete semi-synthesis of full-length cyanobacterial MT. This chemoenzymatic strategy involves SPPS which is thiol-friendly due to the application of thiol scavengers and mild ligation conditions (pH 5.6) and which prevents further oxidation of any (even acidic) cysteine residues. This property of the present method makes it a simple and effective strategy for the synthesis of Cys-containing proteins, especially MTs. The MTs are not just Cys-rich proteins but proteins having special clustering of the cysteines in CC, CxC, and CxxC motifs.^44^ The presence of these clusters could cause interference in other chemical ligation strategies. It should be noticed that one of the main advantages of OaAEP1_C247A ligase, besides its excellent kinetics of catalytic reactions, is its relatively shorter recognition motif, compared to other enzymes, which are widely used for isopeptide bond formation during protein semisynthesis.^35,36^ The presented recognition motifs required by OaAEP1_C247A are NGL in the N-terminal peptide and GL/GV in the C-terminal peptide, but their sequence requirements are not stringent and are wider.^32,35^ The chemoenzymatically synthesized proteins by the present approach will possess a tripeptide motif, Asx-Xaa-Yaa (P1-P1″-P2″), where the P1 residue is the Asn or Asp residue and comes from the N-terminal substrate, while P1″ is non-specific, P2″ is a hydrophobic amino acid, and they come from the C-terminal substrate. Such tripeptide sequences are naturally present or could be very easily introduced artificially to the unstructured regions of many proteins, hence a wide range of proteins and their derivatives can be effectively obtained. The presented protein has 56 amino acid residues, but the approach can be applied in the future for longer proteins due to the fact that longer peptidyl substrates can be obtained not only by the SPPS approach but also expressed and purified in a bacterial system. In combination with recombinant protein expression and other protein engineering enzymes like butelase and sortase, this method could be extended for the synthesis of larger proteins. However, an increase in the substrate’s length may lead to a decrease in the yield of the process. One of the limitations associated with the current method could be its application to protein sequences having more than one recognition motif for the enzymes. However, it is very unlikely that two recognition motifs are present in one protein sequence. As a result, the current experimental work presents a fast, selective, and efficient synthetic strategy to obtain SmtA using recombinant OaAEP1 ligase. It may also provide an excellent path for producing other proteins for biomedical applications, which via other pathways is not effective or still remains impossible, e.g., using NCL.

## Experimental Procedures

### Materials

1,2-Ethanedithiol (EDT), thioanisole, anisole, triisopropylsilane (TIPS), 2-[(2-hydroxy-1,1-bis(hydroxymethyl)ethyl)amino]ethanesulfonic acid (TES), and EDTA sodium acetate were purchased from Sigma-Aldrich. 5,5′-Dithiobis(2-nitrobenzoic acid) (DTNB) was purchased from TCI America. The metal-chelating resin Chelex 100 was from Bio-Rad. *N*,*N*-Dimethylformamide (DMF) and HCl were from VWR Chemicals. Acetonitrile (MECN), HCl (trace metal grade), NaClO_4_·H_2_O, ZnSO_4_·7H_2_O, 4-(2-pyridylazo)resorcinol (PAR), polyethylene glycol (PEG) 3300, and Triton X-100 were from Merck Millipore. Diethyl ether, dichloromethane (DCM), and dimethyl sulfoxide (DMSO) were from Avantor Performance Materials Poland (Gliwice, Poland). TCEP, 1-methyl-2-pyrrolidinone, *N*,*N*,*N*,*N*-tetramethyl-*O*-(1*H*-benzotriazol-1-yl)uranium hexafluorophosphate, trifluoroacetic acid (TFA), *N*,*N*-diisopropylethylamine, piperidine, TentaGel S Ram, Fmoc-Leu-Wang resin, and Fmoc-protected amino acids were obtained from Iris Biotech GmbH (Marktredwitz, Germany). The peptide Dabcyl-YAKGNGL-EDANS was purchased from ProteoGenix. Diethyl ether, acetic anhydride, DMF, DCM, acetonitrile (MeCN), and sodium chloride (NaCl) were from Avantor Performance Materials Poland S.A. Tryptone, yeast extract, Luria–Bertani (LB) broth, agar, agarose, isopropyl-β-d-1-thiogalactopyranoside (IPTG), and sodium dodecyl sulfate (SDS) were purchased from Lab Empire. Ampicillin, chloramphenicol, 1,4-dithiothreitol (DTT), and Tris base were from Roth; pTYB21 vector and chitin resin were from New England BioLabs; Chelex 100 resin was obtained from Bio-Rad, 4-(2-hydroxyethyl)piperazine-1-ethanesulfonic acid sodium salt (HEPES) was from Bioshop, DTNB from TCI Europe N.V., PEG 3350 from Hampton Research, TentaGel R RAM and TentaGel S-NH_2_ resins from Rapp Polymere GmbH. Solvents, including ethanol and DMSO, were purchased from Sinopharm Chemical Reagent Co. Ltd. Aqueous solutions were configured with Milli-Q water (18.2 MΩ cm^–1^, 0.22 μm filter). Microporous membrane filters (0.22 and 0.45 μm) were used for further purification (Jet Biofil, China). All reagents were purchased from commercial suppliers and used as such. All buffers were prepared with Milli-Q water obtained with a deionizing water system (Merck KGaA). To eliminate trace metal ion contamination, all pH buffers were treated with Chelex 100 resin and degassed over 2 h prior to use. For the culture of *E. coli*, LB medium and agar plates were used.

### Peptide Synthesis

All peptides, except Dabcyl-YAKGNGL-Edans, were synthesized via solid-phase synthesis on TentaGel S Ram resin (substitution 0.22 mmol/g) and Fmoc-Leu-Wang resin (substitution 0.22 mmol/g) using the Fmoc strategy and a Liberty 1 microwave-assisted synthesizer (CEM). The reagent excess, cleavage, and purification were performed as previously described.^56,57^ Unless noted otherwise, all reactions were carried out at room temperature. Peptides were cleaved from the resin with a mixture of TFA/anisole/thioanisole/EDT/TIPS (88/2/3/5/2 v/v/v/v/v) over a period of 2.5 h followed by precipitation in cold (−70 °C) diethyl ether. The crude peptide was collected by centrifugation, dried, and purified via RP-HPLC (Varian) on a Phenomenex C18 preparative column, Gemini, 5 μm, 10 × 250 mm, using a gradient of MeCN in 0.1% TFA/water from 1 to 70% over 40 min at a flow rate of 10.0 mL/min. Fractions containing the pure product were collected, identified by an API 2000 Applied Biosystems electrospray ionization (ESI) mass spectrometer and lyophilized. The list of synthesized peptides along with their calculated and observed mass is presented in Table S1. Concentrations of obtained Cys-containing peptides were determined spectrophotometrically at 412 nm using the DTNB assay, and the molar absorption coefficient of the TNB^–^ ion was 14 150 M^–1^ × cm^–1^.^58^ Concentrations of peptides were additionally determined by a NanoDrop A280.

### Expression and Purification of Asparaginyl Endopeptidase (OaAEP1, Mutant C247A)

OaAEP1_C247A is a cysteine 247 to the alanine mutant of asparaginyl endopeptidase 1 (OaAEP1) from *O. affinis*.^59,60^ The plasmid pBHRSF184 was a gift from Hideo Iwai (Addgene plasmid # 89482).^61^ The C247A mutant was generated by site-directed mutagenesis using the following primers:

Fwd. primer: 5′-AGCTGGGCGTATTATTGTCCGGCGCA-3′;

Rev. primer: 5′-AATAATACGCCCAGCTGCTTTCGGTGGTGTTG-3′.

The mutation was confirmed by DNA sequencing. The WT AEP and C247A mutant plasmid DNA was transformed in *E. coli* RIL cells for protein expression. The bacteria cultured in fresh super broth (SB) media with 25 μg/mL kanamycin were induced by IPTG (final concentration, 0.1 mM) for 24 h at 20 °C. The cells were collected by centrifugation at 4000 rpm for 10 min at 4 °C and resuspended in lysis buffer (50 mM Tris/HCl, pH 7.4), 150 mM NaCl, 0.1% Triton X100, and 1 mM EDTA/10% glycerol. The cells were lysed by ultrasonication. After centrifugation at 10 000 rpm for 60 min, the clear supernatants were incubated with Ni(II)-NTA resin and equilibrated with ice-cold lysis buffer. The recombinant proteins were subsequently washed with washing buffer (50 mM Tris/HCl, pH 7.4, 150 mM NaCl, 30 mM imidazole, 0.1% Triton X100, and 1 mM EDTA/10% glycerol) and eluted in elution buffer (50 mM Tris/HCl, pH 8, 150 mM NaCl, 500 mM imidazole, and 1 mM EDTA/10% glycerol). The imidazole was then removed from the elute by size-exclusion chromatography on a PD-10 desalting column using phosphate buffered saline. In the next step, the fusion protein was digested at 24 °C for 2 h by addition of a final concentration of 1 mM DTT and 37 nM Ulp1 protease. Ni(II)-NTA resin was then successfully applied to remove N-terminally His-tagged SMT3 and His_6_-Ulp1 protease from the reaction mixture. The final concentrated protein was purified by SEC. To the collected fraction, 1 mM EDTA and 0.5 mM TCEP were added (final concentration), and the pH of the protein solution was adjusted to 4.0 with glacial acetic acid and incubated at 37 °C for 5 to 16 h for self-cleavage activation. Most of the contaminating proteins precipitated during activation (they were removed by centrifugation). The mature active enzyme was concentrated and stored in aliquots at −80 °C for further usage. Protein concentrations were routinely determined by the Nanodrop 2000 using the respective molecular weight and molar extinction coefficients (Table S1).

### Fluorometric Activity Assay for OaAEP1_C247A Using Dabcyl-YAKGNGL-Edans

The fluorometric activity experiment was performed in 1 mL of reaction mixture containing buffer (50 mM sodium acetate pH 5.6, 50 mM NaCl, 1 mM EDTA, 0.5 mM TCEP), OaAEP1 C247A (25 μM), and 25 mM of Dabcyl-YAKGNGL-Edans. Reactions were initiated by the addition of enzyme and were monitored by measuring the fluorescence spectra from 400 to 600 nm and observing the increase in fluorescence (λ_ex_ = 340 nm, λ_em_ = 490 nm, 37 °C) at different timepoints (Figure 3).

### HPLC-Monitored Model Peptide Activity Assay for OaAEP1_C247A

The assay was carried out in 20 μL of reaction mixture containing buffer (50 mM sodium acetate pH 5.6, 50 mM NaCl, 1 mM EDTA, and 0.5 mM TCEP), OaAEP1 C247A (25 μM), 0.5 mM N-terminal substrate (YKLANGL), and 1 mM C-terminal substrate (GVGKY-NH_2_ or GLGKY-NH_2_). Reactions were initiated by the addition of enzyme and incubated at 37 °C for 2 h. The reaction was quenched by the addition of 80 μL of 0.1% TFA, and the reaction mixtures (final volume, 100 μL) were injected directly onto a C18 analytical RP-HPLC column. The reaction was analyzed using a MeCN/0.1% TFA gradient from 5 to 35% in 40 min and from 35 to 85% in 20 min, and absorbance was recorded at 220 nm. The percentage of substrate converted to product was calculated by integrating the area under the HPLC trace. To confirm the composition and identity of each product, the peaks were collected and analyzed by ESI-MS (Figure 3).

### Expression and Purification of Cyanobacterial MT SmtA

The cDNA encoding MT *S. elongatus* (SmtA) (Uniprot ID: P30331) was synthesized on request by GenScript (USA) and cloned into the pTYB21 vector between SapI and PstI restriction sites using following primers:

Fwd. Primer: 5′-TTTTTTTGCTCTTCCAACATGACCTCGACGACCCTGGTG-3′;

Rev. Primer: 5′-TTTTTTCTGCAGTTAACCGTGACAGTTGCAGCCCG-3′.

Gene inserts were ligated using T4 ligase. The positive clones were confirmed by DNA sequencing. SmtA was obtained as described previously with some minor changes.^49,62^ The pTYB21 vector containing the SmtA gene was transformed into BL21(DE3)pLysS *E. coli* competent cells. The culture medium (1.1% tryptone, 2.2% yeast extract, 0.45% glycerol, 1.3% K_2_HPO_4_, and 0.38% KH_2_PO_4_) was prepared according to Hong et al. and supplemented with 0.3 mM ZnCl_2_.^63^ 10 mL of bacteria preculture was inoculated into each of four flasks with 1 L of medium and cultured until OD_600_ reached 0.6 at 37 °C. The protein expression was induced with 0.1 mM IPTG and incubated overnight at 20 °C with vigorous shaking. Cells were collected by centrifugation at 4000*g* for 15 min at 4 °C, suspended in cold buffer A (20 mM HEPES, pH 8.0, 500 mM NaCl, and 1 mM TCEP) and sonicated three times for 15 min (5 s “on” and 10 s “off“), and this was followed by centrifugation at 16 000*g* for 45 min at 4 °C. The supernatant was loaded onto chitin resin. Incubation was conducted with mild shaking overnight. In the next step, chitin resin in each column was washed six to seven times with buffer A. To start the cleavage reaction, 40 mL of buffer B (20 mM HEPES, pH 8.0, 500 mM NaCl, and 100 mM DTT) was added to the resin, and the mixture was incubated for 36–48 h at room temperature with mild shaking. Thionein was purified on a Superdex 75 Increase 10/300 (Cytiva) in 10 mM HCl. The mass was confirmed using an ESI-quadrupole time-of-flight (QToF) mass spectrometer (compact QToF, Bruker Daltonik GmbH). The DTNB assay was used for the determination of free thiol concentration.^58^ This prepared protein was taken for the experiment or reconstitution was performed using ZnSO_4_ under a nitrogen blanket, and the pH was adjusted to 8.6 with a 1 M solution of Tris base as described previously.^49,64^ MT was concentrated with Amicon Ultra-4 centrifugal filter units, containing a membrane cut-off of 3 kDa (Merck) and purified on a Superdex 75 Increase 10/300 (Cytiva) in 20 mM Tris–HCl pH 8.6. The concentrations of thiolates were determined as before using the DTNB assay.^58^ The concentrations of Zn(II) and protein saturation fraction were determined using the PAR assay at 492 nm using a 71 500 M^–1^ × cm^–1^ molar coefficient for ZnH_*x*_(PAR)_2_.^65^ The Zn(II)-saturated protein was stored at −80 °C. Prior to its use, it was reduced by 1 mM TCEP and acidified using 7% HCl. Then, SmtA was purified on a Superdex 75 Increase 10/300 (Cytiva) in 10 mM HCl.^49,50^

### Spectroscopic Titrations of Metal-free SmtA (Apo-SmtA) with Zn(II) and Cd(II)

Spectrophotometric measurements were recorded using a JASCO V-650 spectrophotometer at 25 °C in a 1 cm quartz cuvette over the UV range of 200–300 nm. 1 μM freshly prepared thionein (in 0.01 M HCl) was used for the experiment. Titrations with Zn(II) and Cd(II) were performed in 50 mM chelexed borate buffer pH 7.4 (0.1 M NaClO_4_). 0.5 mM ZnSO_4_ or CdSO_4_ stock solutions were added in small portions, increasing the metal concentration by about 0.5 equiv.^49^ The samples were incubated over 1 min after the addition of each portion of the metal solution. The titrations were made to a final metal-to-protein ratio of 8. TCEP was added as a weakly metal-binding reducing agent to at least 5 M equiv excess (final concentration, 45 μM) over each cysteine thiol, and all titrations were performed under an argon atmosphere.^66,67^

### Circular Dichroism

Circular dichroism (CD) spectra were recorded using a J-1500 JASCO spectropolarimeter at 25 °C in a 2 mm quartz cuvette under a constant nitrogen flow over the range of 200–260 nm with a 100 nm/min speed scan. 20 μM metal-free and SmtA with Zn(II) were measured in 10 mM Tris–HCl buffer (100 mM NaClO_4_, pH 7.4). 10 mM TCEP (pH 7.4) was added to a final concentration of 360 μM as a weak-metal-binding cysteine thiol protector. Samples were equilibrated over 2 min after the addition of ZnSO_4_ solution prior to recording CD spectra.

### Spectrophotometric pH-Titration of SmtA with Zn(II)

The spectrophotometric measurements were recorded using a JASCO V-650 spectrophotometer at 25 °C in a 1 cm quartz cuvette. 1 μM of the freshly prepared thionein (in 0.01 M HCl) was used for the experiment. Then, 4.2 excess of 0.5 mM ZnSO_4_ was added. Thionein was titrated with incremental amounts of 0.05–2 M NaOH in a pH range from 3 to 8. The spectra were recorded in the wavelength range of 200–300 nm. Dilution effects due to NaOH addition are negligible.

### Ligation of N- and C-Terminal Substrates of SmtA and Its Optimization

100 μM asparaginyl endopeptidase was incubated with 0.5 mM N-terminal substrate and 0.25 mM C-terminal peptide substrate in 20 μL of assay buffer (50 mM sodium acetate pH 5.6, 50 mM NaCl, 1 mM EDTA, and 0.5 mM TCEP) at 37 °C for different time periods. The reactions were quenched by adding 80 μL of 0.1% TFA and injected (final volume = 100 μL) onto a C18 RP-HPLC column. Peptides were eluted by applying a gradient from 5 to 35% in 40 min, from 35 to 85% MeCN/0.1% TFA in 20 min at a flow rate of 1 mL/min. Absorbance was recorded at 220 nm. Peak fractions were collected, and their identities were confirmed by ESI-MS. The amount of product produced and the N-terminal substrate conversion value were determined by integrating the area of the product peak in the HPLC trace. Additionally, different molar ratios of N- and C-terminal substrates were tested in order to investigate the influence of the substrate ratio on the product formation rate: 1:1 (0.25 and 0.25 mM), 1:2 (0.25 and 0.50 mM), and 2:1 (0.5 and 0.25 mM), in the presence of 100 μM OaAEP1 ligase at 37 °C for 1 h (Figures 4 and S2). The identity of the peaks was confirmed by ESI-MS.

### Semi-preparative Production and Purification of SmtA Using Size Exclusion Chromatography

Size exclusion chromatography (SEC)/fast protein liquid chromatography was applied as a preparative method in order to collect larger amounts of the synthetic product. 100 μM asparaginyl endopeptidase was incubated with 0.5 mM N-terminal and 0.25 mM C-terminal peptidyl substrates in 200 μL of assay buffer (50 mM sodium acetate pH 5.6, 50 mM NaCl, 1 mM EDTA, and 0.5 mM TCEP) at 37 °C for 1 h. The reactions were quenched and subjected to SEC on a Superdex 75 Increase 10/300 (Cytiva) in 10 mM HCl. Absorbance was recorded at 220 and 280 nm. The product peak was collected and incubated with 100 mM DTT for 2 h at room temperature. It was then acidified using 7% HCl (v/v) to a final pH 2.2 and then concentrated using 3 kDa Amicon Ultra-4 centrifugal filter units. The apo-protein was again purified on a SEC-70 gel filtration column (Bio-Rad) equilibrated with 10 mM HCl. To confirm the composition and identity of the product, the product peak was collected and analyzed by ESI-MS (Figures 5 and S4).

## Supporting Information Available

Molecular masses of peptides and proteins, SDS gel profile of the purified OaAEP1_C247A enzyme, optimization of reaction conditions for the enzymatic ligation of SmtA, RP-HPLC and SEC profiles for peptides and the monitored reaction, differential absorption spectra representing metal titrations, and CD spectra (PDF)
