# Supplementary material for: OaAEP1 Ligase-Assisted Chemoenzymatic Synthesis of Full Cysteine-Rich Metal-Binding Cyanobacterial Metallothionein SmtA
Source: Bioconjug Chem. 2023 Mar 15;34(4):719–27. doi: 10.1021/acs.bioconjchem.3c00037 (PMC10119931; doi:10.1021/acs.bioconjchem.3c00037)
Supplement: Supplementary file 1 — bc3c00037_si_001.pdf [file bc3c00037_si_001.pdf]

Supporting information

OaAEP1 ligase-assisted chemoenzymatic synthesis of full cysteine-rich metal-binding cyanobacterial metallothionein SmtA

Anastasiia Antonenko, Avinash Kumar Singh, Karolina Mosna and Artur Krężel*

Department of Chemical Biology, Faculty of Biotechnology, University of Wrocław, Joliot-Curie 14a, 50-383 Wrocław, Poland

*Correspondence to A. Krężel, E-mail: artur.krezel@uwr.edu.pl

Table S1. Experimental and expected molecular masses of peptides and proteins synthesized in this study. Dabcyl and Edans refer to *N*-[4-(4-dimethylamino)phenylazo]benzoic acid and 5- ((2-aminoethyl)amino)naphthalene-1-sulfonic acid, respectively.

Peptide/protein sequence	Experimental mass average (Da)	Expected mass average (Da)	Molar absorption coefficient (M^{-1} cm^{-1})[a]
GGGKY-NH_2	479.68	480.52	1490
YKLANGL	777.81	777.92	1490
YKLANGGGKY-NH_2	1069.87	1070.21	2980
YKLAN	607.23	607.71	1490
Dabcyl-YAKGNGL-Edans	1220.95	1221.39	11100
MTSTTLVKCACEPCLCNVDPSKAIDRNGL	3082.42	3083.62	250
GLYYCSEACADGHTGGSKGCGHTGCNCHG	2843.97	2846.07	2980
MTSTTLVKCACEPCLCNVDPSKAIDRN	2913.89	2913.41	250
bacterial SmtA	5740.77	5741.46	2980
synthesized SmtA	5740.22	5741.46	2980

[a]Molar extinction coefficients were calculated using ExPASy ProtParam tool, https://web.expasy.org/cgi-bin/peptide_mass/peptide-mass.pl.

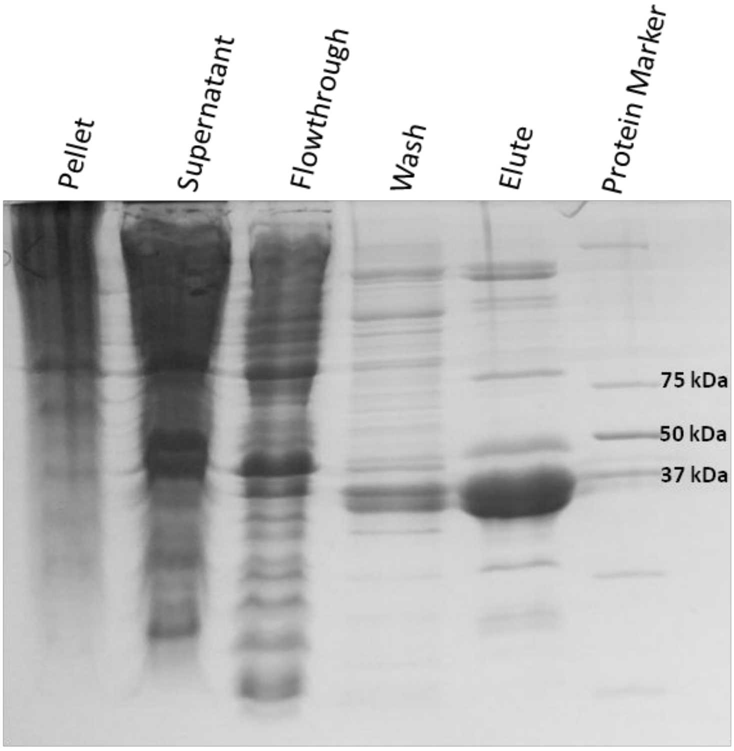

Figure S1. SDS gel profile of purified OaAEP1_C247A enzyme (MW 33,846.34 Da). It was expressed and purified from *E. coli* by Ni-NTA affinity purification followed by desalting on PD-10 columns.

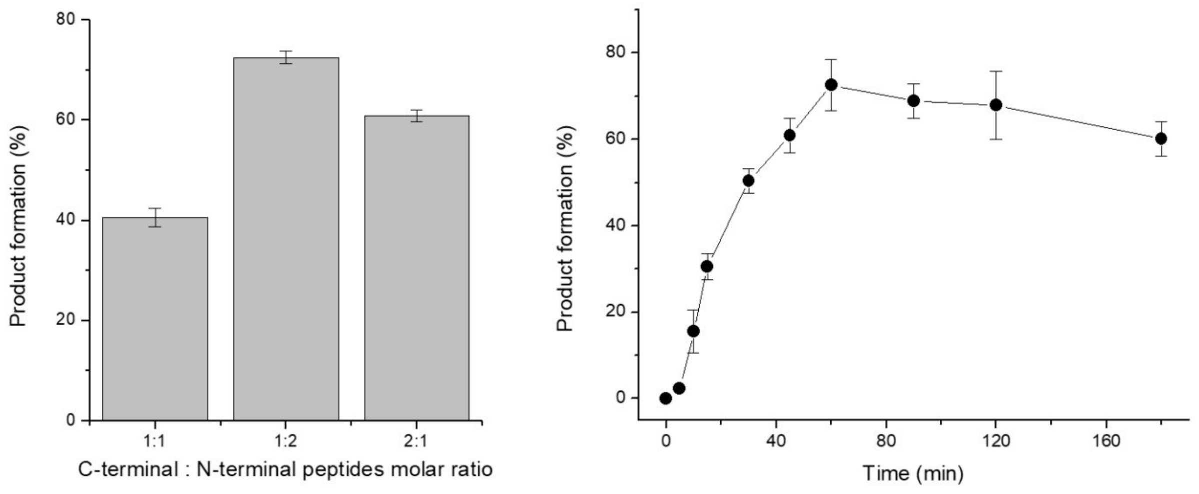

Figure S2. Optimization of reaction conditions for ligation of SmtA N-terminal and C-terminal substrates with OaAEP1. A) Influence of substrate ratio on product formation. Each point represents the mean value ± standard deviation (n = 3). Ligation was carried out using different molar ratio of C-terminal peptide as a model donor and N-terminal peptide as an acceptor peptide, 1:1 (0.25 mM and 0.25 mM), 1:2 (0.25 mM and 0.50 mM) and 2:1 (0.5 mM and 0.25 mM), accordingly, in the presence of 100 μM OaAEP1 ligase at 37°C for 1 h. B) Time-dependent product formation curve for the ligation reaction. Ligation was carried out using 0.5 mM N-terminal substrate and 0.25 mM C-terminal substrate in the presence of 100 μM OaAEP1 C247A at 37°C.

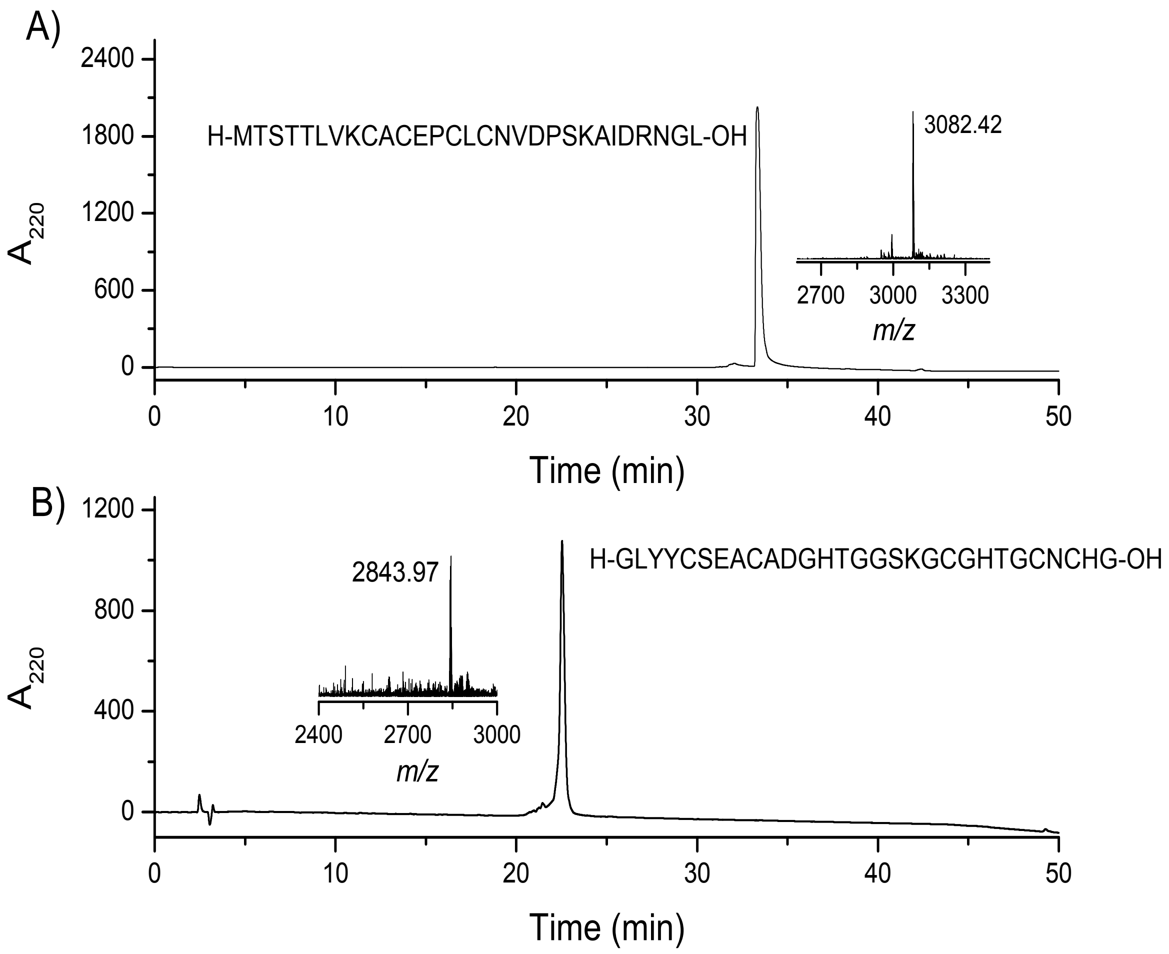

Figure S3. RP-HPLC chromatograms of N-terminal (A) and C-terminal (B) substrates of SmtA ligation on a C18 column (250 mm × 4.6 mm, 5.0 µm) in a gradient 5–35% of MeCN in 40 minutes, 35–85% of MeCN in the next 20 minutes and absorbance was recorded at 220 nm.

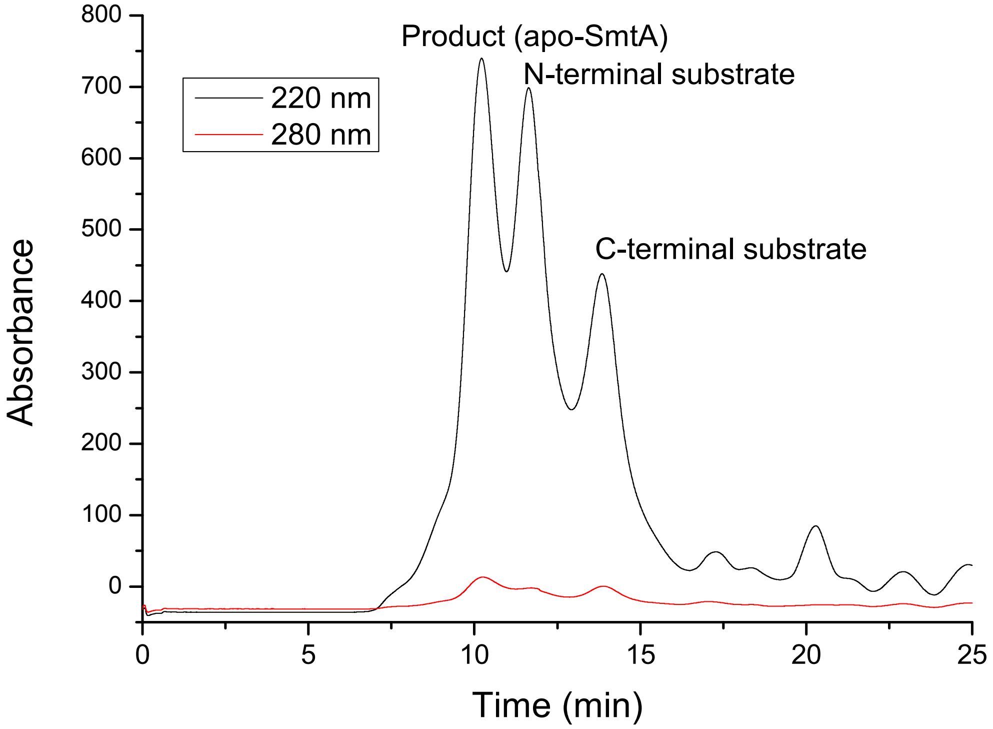

Figure S4. SEC reaction profile of 0.5 mM N-terminal and 0.25 mM C-terminal substrates in the presence of 100 μM OaAEP1 C247A at 37°C in 1 h. Analysis was done on a Superdex Peptide column (10/300 GL) using 10 mM HCl and absorbance was recorded at 220 and 280 nm. The peak for the hydrolyzed product was found to overlap with the peak of N-terminal substrate.

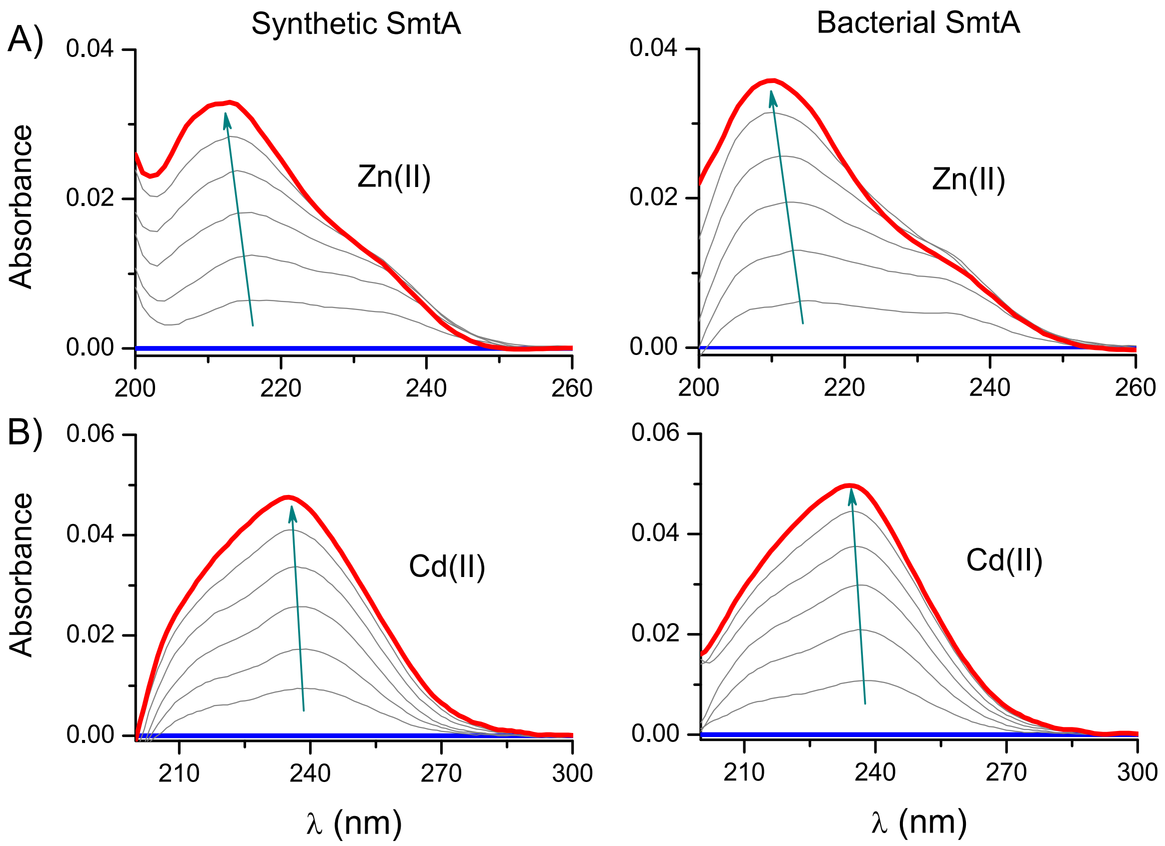

Figure S5. Differential absorption spectra representing Zn(II) (top row) and Cd(II) (bottom row) titrations presented in Figure 4. Left panel corresponds to Zn(II) and Cd(II) titrations of chemoenzymatically synthesized apo-SmtA. Right panel shows Zn(II) and Cd(II) titrations of apo-SmtA produced in the *E. coli* system. Green arrows demonstrate the change of maxima bands upon metal addition.

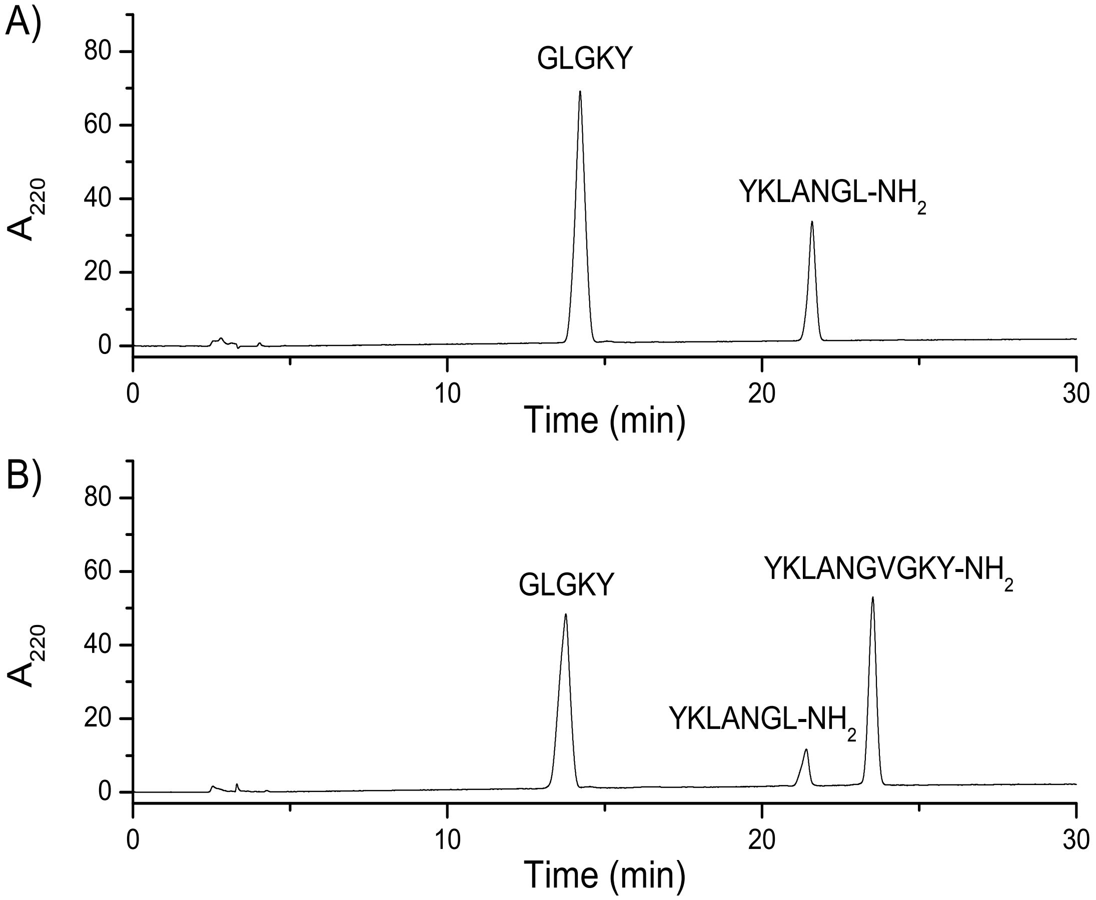

Figure S6. RP-HPLC-monitored ligation of model peptides, N-terminal YKLANGL and C-terminal GLGKY-NH_2 using OaAEP1 ligase. A) HPLC profile of 0.25 mM N-terminal and 0.5 mM C-terminal peptides. B) HPLC profile of peptide mixture with 25 µM ligase monitored during reaction at 37°C after 2 h. Hydrolysed product (YKLAN) elutes with the GLGKY. Peptides (substrates and products) were separated on a C18 column (250 mm × 4.6 mm, 5.0 µm) in a gradient 5-35% of MeCN in 40 minutes, 35–85% of MeCN in the next 20 minutes and absorbance was recorded at 220 nm.

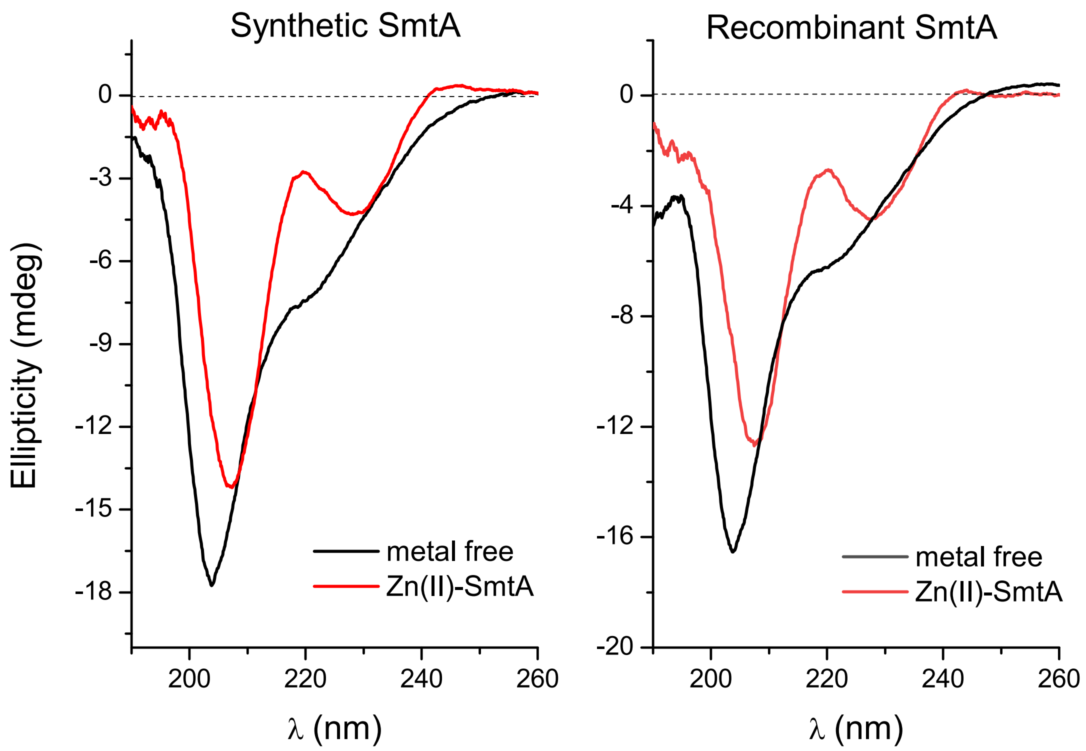

Figure S7. CD spectra of metal-free and Zn(II) loaded SmtA chemosynthesized (A) and obtained in E. coli (B). 20 μM metal-free and SmtA with Zn(II) measured in 10 mM Tris-HCl buffer (100 mM $NaClO_4$, pH 7.4). 10 mM TCEP (pH 7.4) was added to a final concentration of 360 μM as a weak-metal binding cysteine thiol protector.
